# Investigation of the validity, reliability and psychometric properties of the Turkish version of the Ottawa sitting scale in patients with Parkinson’s disease

**DOI:** 10.1038/s41598-025-87006-7

**Published:** 2025-01-21

**Authors:** Aysu Yetiş, Mehmet Canli, Nazım Tolgahan Yildiz, Hikmet Kocaman, Hasan Yildirim, Şafak Kuzu, İrem Valamur, Selcen Duran

**Affiliations:** 1 Faculty of Medicine, Department of Neurology, Kırşehir Ahi Evran University, Kırşehir, Turkey; 2 School of Physical Therapy and Rehabilitation, Kırşehir Ahi Evran University, Kırşehir, Turkey; 3 Faculty of Health Sciences, Deparment of Physiotherapy and Rehabilitation, Karamanoglu Mehmetbey University, Karaman, Turkey; 4 Faculty of Kamil Özdağ Science, Department of Mathematics, Karamanoğlu Mehmetbey University, Karaman, Turkey

**Keywords:** Sitting balance, Ottowa sitting scale, Parkinson's disease, Validity, Reliability, Diseases, Medical research

## Abstract

The Ottawa Sitting Scale is a tool for the multidimensional assessment of sitting balance. This study aimed to investigate the validity, reliability, and psychometric properties of the Turkish version of the Ottawa Sitting Scale (OSS-TR) in Turkish-speaking patients with Parkinson’s disease (PD). The study included 56 patients diagnosed with PD based on the UK Brain Bank Criteria. Construct validity of the OSS-TR was established through the evaluation of structural and convergent validity. Explanatory factor analysis and confirmatory factor analysis (CFA) were carried out to determine the structural validity. Convergent validity was analysed by examining the relationships between OSS-TR with the Berg Balance Scale (BBS) and Trunk Impairment Scale (TIS). The test-retest reliability of the scale was assessed by intraclass correlation coefficient (ICC) and internal consistency was assessed by Cronbach’s alpha coefficient. The good fit determined according to the model fit criteria based on the CFA results confirmed the structural validity. Furthermore, the high associations between OSS-TR with the BBS (*r* = 0.766) and TIS (*r* = 0.720) supported convergent validity (*p* < 0.05). Test-retest reliability of the OSS-TR was excellent (ICC = 0.867). Moreover, internal consistency was high (Cronbach’s alpha = 0.948). The OSS-TR is a valid and reliable instrument for assessing sitting balance in Turkish-speaking PD patients. Regarding the results of the study, OSS-TR can be considered useful in the evaluation of sitting balance among PD patients in clinical and research settings.

## Introduction

Parkinson’s disease (PD) is one of the most prevalent neurodegenerative disorders, affecting over 6 million individuals globally^1^. In PD, both motor and non-motor dysfunctions arise from the destruction of dopaminergic neurons. The most common symptoms include impairments in speech, gait, posture, balance, and cognitive functions^2,3^. These symptoms are often interrelated and significantly diminish the quality of life for patients with PD^4,5^. The global prevalence of PD ranges from 15 to 657 per 100,000 individuals^6^. In Turkey, the prevalence is approximately 202 per 100,000 individuals^1^.

Balance refers to the ability to maintain normal body posture while sitting, standing, or moving. It is crucial for positioning the body appropriately during various activities and in response to environmental changes. Impairments in balance control can result in falls, gait disturbances, and reduced mobility, leading to a loss of functional independence and social isolation in daily life^7^.

The underlying mechanism of balance impairment in PD patients is believed to be a malfunction of sensory organization involving visual, vestibular, and somatosensory systems^7^. A review of the literature reveals that most PD patients experience balance disorders shortly after disease onset^8,9^, with approximately one-third developing balance disorders within the first two years following diagnosis^10^. Sitting balance, a crucial component of postural control, is essential for engaging in activities of daily living. Adequate sitting balance enables individuals with neurological diseases to participate safely and effectively in these activities^11^. There is no specific measurement method in the literature for assessing sitting balance in neurological diseases. However, the Unified Parkinson’s Disease Rating Scale is used to evaluate postural instability in PD patients, although it includes only one item that assesses postural instability^12^.

The assessment of sitting balance in PD patients using a practical tool is crucial for developing treatment programs and evaluating treatment efficacy. The Ottawa Sitting Scale (OSS) is a concise measurement tool consisting of 10 items designed to assess sitting balance, encompassing both static and dynamic aspects^13^. Unlike other balance assessment tools used in neurological diseases, the OSS focuses exclusively on sitting balance^13^. The validity, reliability, and psychometric properties of the Turkish version of the OSS (OSS-TR) have been previously examined in patients with acute stroke^14^and those discharged from the intensive care unit^15^, concluding that it is a valid and reliable tool for assessing sitting balance.

The validity, reliability, and psychometric properties of the OSS-TR have not been analyzed in PD patients. Demonstrating the validity and reliability of the OSS-TR in this population will provide valuable information for examining the effect of PD on sitting balance. Therefore, the aim of this study was to investigate the validity, reliability, and psychometric properties of the OSS-TR in Turkish-speaking PD patients.

## Materials and methods

### Study design and ethical aspects participants

The approval of the local ethics committee was obtained to conduct this cross-sectional study (Number: 2024-12/93). The study was performed in accordance with the ethical standards as laid down in the Declaration of Helsinki and its later amendments. This study was conducted following COSMIN guidelines^16^. Written and verbal informed consent was obtained from the participants before the study.

## Participants

The study sample consisted of patients who were admitted to the Neurology Clinic of Kırşehir Training and Research Hospital, whose native language was Turkish, and who were diagnosed with PD by a neurologist according to the UK Brain Bank Criteria^17^. Other inclusion criteria were as follows: To be over 18 years of age, to have a score of 24 or above on the Mini-Mental State Test and not to have any cognitive abnormalities. Patients with an additional neurological disorder other than PD, having vestibular disorders, and having a history of medication or musculoskeletal system disorders that may affect balance were not included in the study.

## Sample size

Although there is no specific technique for determining the sample size in the studies conducted to examine the validity, reliability, and psychometric properties of the questionnaire, the common procedure suggests that a sample size of 5–10 participants should be reached for each item of the questionnaire^18^. This method has been used and recommended for sample size calculation in previous psychometric studies based on the COSMIN checklist^18,19^. Considering that the OSS-TR consists of 10 items and with reference to previous studies, it was considered appropriate to conduct this study with more than 50 participants^18–20^.

## Procedures

The necessary permissions were received from the authors of the OSS-TR before initiating the study. To determine the test-retest reliability of the OSS-TR, the instrument was re-administered to patients with PD by the same examiner with an interval of 7 days. Demographic and clinical characteristics of the subjects were documented. The Turkish versions of the TIS and BBS were also implemented to the participants to analyse the convergent validity of the OSS-TR.

## Outcome measures

### Ottowa sitting scale

The OSS-TR was used to assess the sitting balance of the PD patients^14^. The difference of the scale from other balance scales is that it evaluates sitting balance specifically and can easily evaluate sitting balance in patients without ambulation ability. The original OSS was developed to assess sitting balance in acute care patients^13^. The OSS-TR consists of 10 items assessing sitting balance in foot-supported and foot-unsupported positions. The scale assesses the ability to maintain a static sitting position, the ability to reach in different directions while sitting, the ability to rotate the trunk in a stable sitting position, and the ability to move forwards and backwards in bed by means of pelvic movements. Each item is levelled from 0 (worst) to 4 (best). A low score indicates a more severe impairment of the sitting balance ability^13^.

## Berg balance scale

The BBS assesses an individual’s ability to maintain balance during functional activities. The test consists of 14 items, and each item is graded between 0 and 4. 0 indicates the worst score, and 4 indicates the best score. The highest score that can be reached in total is 64 points. A higher score represents a better balance^21^.

## Trunk impairment scale

The TIS, which was developed by Verheyden et al^22^. and validated in Turkish^23^, was used for the evaluation of trunk control. The scale consists of 3 subsections and 17 items measuring static and dynamic sitting balance as well as coordination. In the TIS, where a total score between 0 and 23 is obtained, a high score means that balance and trunk control are good^23^.

### Statistical analysis and methodology

The statistical analyses of the study were conducted via RStudio^24^based on R language v. 4.3.2, lavaan^25^and semPlot packages^26^, IBM SPSS 24.0 (IBM Corp.). Statistical significance was set at *p* < 0.05. The numerical variables were summarized with mean and standard deviation, whereas the categorical variables were represented by percentage and frequency values. The conformity of the variables in the data set to a normal distribution was analyzed by analytical and visual methods. Pearson correlation analysis was employed to evaluate the associations between OSS-TR score with BBS and TIS scores.

### Construct validity

Construct validity refers to the extent to which a test or questionnaire accurately measures the concept it is intended to assess. Essentially, it evaluates how well the items within the test or questionnaire represent the target concept. In this study, the construct validity of the OSS-TR was examined by assessing both its structural and convergent validity^27^.

### Structural validity

In this study, the structural validity, representing the degree to which the underlying structure of a measurement instrument accurately reflects the theoretical construct or concept intended to be measured, was assessed via both explanatory factor analysis (EFA) and confirmatory factor analysis (CFA). In EFA conducted prior to confirmatory factor analysis, the weighted least squares (WLS) and direct oblimin rotation methods were implemented to extract factors and obtain a simpler factor structure interpretation, respectively. Kaiser-Meyer-Olkin (KMO) and Bartlett’s test of sphericity were performed to determine whether the data were appropriate for factor analysis^28^. CFA was performed to determine whether the predetermined latent structure of the OSS-TR fit the data collected from Turkish patients with PD. WLS estimate was employed in the CFA, and the standardization factor load threshold of 0.50 or above was used. Tucker-Lewis index (TLI, > 0.90), comparative fit index (CFI, > 0.90), non-normed fit index (NNFI, > 0.90), chi-square statistic according to degrees of freedom (χ^2^2/df, ≤ 2), McDonald fit index (MFI, > 0.90), root mean square error of approximation (RMSEA, < 0.080), standardized root mean square residual (SRMR, < 0.05) indices were calculated for the fit examination of the structure^28^.

### Convergent validity

The convergent validity reflects the degree of relationship among a test and other assessment instruments measuring similar (or identical) constructs. For this purpose, the level of correlation between scales described and validated for similar objectives is assessed via Pearson correlation analysis. In this context, the relationships between the OSS-TR with BBS and TIS were investigated. A correlation value of 0.60 and above indicates a strong validity^18^. Additionally, the average variance extracted (AVE) and construct reliability (CR) were utilized to enhance the assessment of convergent validity. The AVE and CR values greater than 0.50 and 0.70 respectively in the overall scale are indicators of validity^29^.

### Reliability

In order to assess the test-retest reliability of the OSS-TR, 56 patients were re-implemented 7 days after the first administration. The ICC_(2,1)_corresponding to the two-way mixed-effects model of single measure for absolute agreement was calculated according to the obtained measurements and reported with a 95% confidence interval. ICC values were categorized as i) poor (0.00–0.25), (ii) fair (0.26–0.50), iii) moderate (0.51–0.75) and iv) good (0.76–1.00)^30^. The internal consistency of the OSS-TR was assessed by calculating Cronbach’s alpha coefficient for the overall scale. A Cronbach’s alpha coefficient of 0.80 and above implies that the instrument has good internal consistency^31^.

## Results

Of the 60 patients who were diagnosed with PD and referred to our clinic for evaluation, four patients did not fulfil the inclusion criteria and the study was completed with 56 patients. The descriptive statistics of the demographic characteristics of the participants are presented in Table 1. According to these results, the mean age was 64.55 ± 4.85 years, the mean BMI was 25 ± 1.42. The majority of the participants were male (57.1%), and the highest number of participants in terms of Hoehn & Yahr Stage was in the 2.00 group with 16 participants (28.6%).

**Table 1:** Demographic and clinical characteristics of the participants (*n* = 56).

Quantitative features		Mean$\:\pm\:$SD	
Age (years)		64.55 ± 4.85	
BMI (kg/m^2^)		25 ± 1.42	
Disease duration (years)		7.38 ± 1.36	
OSS-TR score (point)		18.90 ± 4.13	
OSS-TR Retest score (point)		16.68 ± 3.61	
Berg Balance Scale score (point)		24.90 ± 3.36	
Trunk Impairment Scale score (point)		11.38 ± 1.94	
		**Count**	**%**
Gender	Male	32	57.1
	Female	24	42.9
Hoehn & Yahr Stage	1.00	13	23.2
	1.50	14	25.0
	2.00	16	28.6
	2.50	9	16.1
	3.00	4	7.1

SD: Standard deviation, BMI: Body mass index, OSS-TR: Turkish version of the Ottawa Sitting Scale.

As the model fit criteria based on the CFA results given in Table 2, χ^2^/df = 1.11, GFI = 0.940, NFI = 0.904, CFI = 0.989, MFI = 0.957, TLI = 0.985, RMSEA = 0.053 and SRMR = 0.035 were utilized and a good fit was determined. During the CFA process, the modification was implemented between some items (1–9, 2–5 and 4–6) by using error covariances. Examining the factor loadings of the items, it was observed that the smallest value was 0.701 and the largest value was 0.903. As neither value was less than 0.50, it was concluded that all of the items should remain in the model and no item should be eliminated. Each parameter estimation was found as statistically significant (*p* < 0.001). According to the KMO analysis, the sample size and observed correlation coefficients and partial correlation coefficients are suitable for factor analysis (Overall MSA = 0.878), and the correlation pattern is sufficient for factor analysis according to Bartlett’s test of sphericity (χ^2^ = 323.670; *p* < 0.001) (Table 2).

**Table 2:** Results of EFA and CFA with model assesment criteria of the OSS-TR.

		Model results for EFA		Model fit indices for CFA		
Item	Factor loadings for CFA	Examination	Values	Indice	Threshold	Values
1	0.701	KMO measure of sampling adequacy	Overall MSA = 0.878	χ^2^/df	≤ 2	1.111
2	0.766			GFI	> 0.90	0.940
3	0.706			NNFI	> 0.90	0.904
4	0.826	Bartlett’s test of sphericity	χ^2^ = 323.670; *p* < 0.001	CFI	> 0.90	0.989
5	0.903			MFI	> 0.90	0.957
6	0.765	Ratio of variance explained	0.654; *p* < 0.001	TLI	> 0.90	0.985
7	0.762			RMSEA	< 0.080	0.053
8	0.830			SRMR	< 0.05	0.035
9	0.782					
10	0.792					

EFA: Exploratory factor analysis, CFA: Confirmatory factor analysis, OSS-TR: Turkish version of the Ottawa Sitting Scale, KMO: Kaiser-Meyer-Olkin, MSA: Measure of sampling adequacy, χ^2^/df: The ratio of the chi-square statistic to degrees of freedom, NNFI: Non-normed fit index, GFI: Goodness of fit index, RMSEA: Root mean square error of approximation, SRMR: Standardized root mean square residual, CFI: Comparative fit index, MFI: McDonald fit index, TLI: Tucker-Lewis index.

The convergent validity results calculated within the overall OSS-TR scale are summarized in Table 3. There were statistically significant, positive and strong correlations between OSS-TR with BBS (*r* = 0.766, *p* < 0.001) and TIS (*r* = 0.820, *p* < 0.001) scores. Considering the AVE and CR criteria, the AVE score (0.617) was greater than 0.50 and the CR score (0.841) was greater than 0.70 on the overall scale, which supported convergent validity.

**Table 3:** Convergent validity of the OSS-TR.

		OSS-TR score
Convergent validity	Berg Balance Scale score	*r* = 0.766 (*p* < 0.001)
	Trunk Impairment Scale score	*r* = 0.820 (*p* < 0.001)
	AVE	0.617
	CR	0.841

OSS-TR: Turkish version of Ottowa Sitting Scale, r: Pearson correlation coefficient. AVE: Average variance extracted, CR: Construct reliability.

Table 4 provides the separate dstatistics and correlation values of the items of the OSS-TR. Based on these results, the corrected total-item correlation statistic for all items was found to be 0.705 and above, and it was observed that it was more appropriate not to exclude any item.

**Table 4:** The summary statistics of the OSS-TR items.

Item	Mean ± SD	Corrected Total-Item Correlation	Cronbach’s α if Item Deleted
1	1.73 ± 0.96	0.752	0.924
2	1.93 ± 0.97	0.771	0.943
3	1.88 ± 1.02	0.705	0.937
4	1.85 ± 0.98	0.805	0.932
5	1.85 ± 1.05	0.823	0.941
6	2.00 ± 1.04	0.711	0.926
7	1.88 ± 0.88	0.84	0.941
8	2.00 ± 0.96	0.852	0.930
9	1.90 ± 1.01	0.794	0.942
10	1.93 ± 0.97	0.807	0.922

SD: Standard deviation, OSS-TR: Turkish version of the Ottawa Sitting Scale.

The results related to the test-retest reliability and internal consistency the OSS-TR were shown in Table 5. The results revealed that the scale has an excellent level of internal consistency (Cronbach alpha = 0.948) and the items stand for the similar construct. Furthermore, OSS-TR’s the test-retest reliability based on ICC was found to be quite high (ICC = 0.867).

**Table 5:** Results of internal consistency and test-retest reliability of the OSS-TR.

		Total Scale
Internal consistency	Cronbach Alpha	0.948
Test-retest reliability	ICC (95% CI)	0.867 (0.768–0.930)

OSS-TR: Turkish version of the Ottawa Sitting Scale, ICC: Intraclass correlation coefficient, CI: Confidence interval.

## Discussion

The present study revealed that the OSS-TR can be used as a valid and reliable instrument to assess sitting balance in PD patients. The internal consistency and test-retest reliability of the scale were high. Additionally, the OSS-TR was strongly correlated with the BBS and TIS, which were frequently used in balance assessments in clinical and research settings.

To maintain postural control against gravity, the ability of sitting balance is required to be good^14^. Sitting balance, which is an important indicator of postural control, affects the participation of PD patients in daily living activities. Patients with neurological disorders such as PD who have good sitting balance and, thus, good postural control can safely and effectively participate in daily living activities^11^. However, sitting balance involves not only a static sitting position, but also the capacity to reach in various directions while sitting^32^. Therefore, measurement tools that assess sitting balance should also evaluate the ability to maintain balance during reaching activities in the sitting position. The OSS was originally developed to assess not only static sitting balance but also sitting balance during reaching activities in various directions while sitting^13^.

Assessment of sitting balance is very important for patients with limited standing ability, low mobility, and who have difficulty performing unassisted sitting activities^13^. BBS and TIS are among the most frequently used instruments to assess balance for neurological diseases in clinical settings^33,34^. However, since these tools do not specifically assess sitting ability, they are insufficient to evaluate the effect of rehabilitation programs on sitting balance.

In the original OSS, Thornton et al^13^. concluded that the scale had a two-factor structure. However, they stated that, contrary to what was thought, items with and without foot support had no effect on factor loading. In the study conducted by Aktaş et al^15^. in which the OSS-TR was implemented in patients with intensive care unit survivors, it was found that items 3,4,5,8,9,10 were included in factor 1 and items 1,2,6,7 were included in factor 2. In the other study conducted in patients with acute stroke^14^, it was concluded that the items of the OSS-TR constituted a two-factor structure, similar to the study of Aktaş et al^15^.. In the present study, the good fit determined according to the model fit criteria based on the CFA results confirmed the structural validity of the OSS-TR in patients with PD. On the other hand, in this study, unlike the original study of the OSS and the version studies in other disease samples, the OSS-TR was found to be single-factor. This result may have been caused by different levels of impairment in the sitting balance of patients in various disease groups.

Measurement tools such as BBS, TIS, Functional Independence Measurement (FIM), and the Physiotherapy Functional Mobility Profile (PFMP) were used to evaluate postural control, balance, and trunk performance in PD patients^13,14,35^. In the present study, BBS and TIS were used to assess the convergent validity of the OSS-TR in patients with PD, similar to the OSS-TR’s previous psychometric studies conducted in other neurological disease groups^14,15^. Since the Turkish version of the PFMP that was used in the original OSS study^13^was not available, the PFMP could not be utilised in this study. Furthermore, AVE and CR values were computed to support the convergent validity of the OSS-TR. In the current study, which found strong associations between OSS-TR with both BBS and TIS in PD patients, it was also observed that AVE and CR values were greater than 0.50 and 0.70, respectively. These results support that the OSS-TR has high convergent validity in PD patients. The confirmation of both structural and convergent validity of the OSS-TR proved that the instrument is constructively valid. In the original OSS study, Thornton et al^13^. reported a moderate relationship between OSS and PFMP in acute care patients. Aktaş et al^15^. showed that there was a strong relationship between OSS-TR and BBS and a moderate relationship between OSS-TR and FIM in patients with intensive care unit survivors. In another study, Yaşa et al^14^. revealed a strong associations between OSS with both BBS and TIS in patients with acute stroke.

Internal consistency is an indicator of the extent to which all items in a multi-item assessment system measure the same construct. It also assesses the variability in performance across different items of a test or outcome measure and is a measure of reliability^36^. The Cronbach’s α coefficient is employed to evaluate the internal consistency of scales. A Cronbach’s α coefficient value of ≥ 0.70 in scale development studies and ≥ 0.80 in the adaptation of previously developed scales to different languages and populations indicate good internal consistency^31^. Considering the Turkish psychometric studies of the OSS-TR, Yaşa et al^14^. reported that the Cronbach α coefficient of the OSS-TR was 0.980 in patients with acute stroke, and Aktaş et al^15^. showed that the Cronbach α coefficient was 0.998 in patients discharged from intensive care. In this study, the Cronbach α coefficient of OSS-TR in patients with PD was determined to be 0.948, in parallel with previous studies. Also, the corrected total-item correlation statistic for all items was found to be 0.705 and above, and it was determined that no item needed to be removed from the scale. These results revealed that the OSS-TR had high internal consistency in patients with acute stroke and patients discharged from intensive care, as well as in patients with PD.

Test-retest reliability refers quantitatively to the consistency of a test or measurement. The most prevalent measure of test-retest reliability is the ICC^37^. In the original OSS study, the ICC value of the scale was found to be 0.994^13^, while the ICC value was found to be 0.998 in patients discharged from intensive care units^15^and 0.996 in patients with acute stroke^14^. These findings indicated that the OSS has high test-retest reliability in different types of diseases. In the current study, the ICC value for the test-retest reliability of the OSS-TR in patients with PD was calculated to be 0.867. Considering the results of the present study, which are consistent with the studies in the literature, it was concluded that the OSS-TR has a high test-retest reliability in patients with PD.

One of the other notable findings of this study is that none of the PD patients scored a minimum (0 point) or maximum score (40 point) on the OSS-TR. Based on these findings, it can be emphasised that the OSS-TR does not have a ceiling or floor effect in PD patients. It was noted that in the original OSS study, only 3 participants scored a minimum score, which was negligible in the floor effect^13^. In the study investigating the OSS-TR in acute stroke patients, it was stated that there was no ceiling or floor effect^14^, while in another study conducted in patients discharged from the intensive care unit, it was reported that the OSS-TR had a ceiling effect, and approximately 18.5% of the participants scored maximum points^15^.

This study has several limitations. Firstly, the study was conducted with a relatively small sample size. Secondly, the convergent validity of the OSS-TR could not be examined with a measurement instrument that specifically measures sitting balance. Thirdly, since this study was planned as a cross-sectional study, the responsiveness of OSS-TR could not be analyzed. Lastly, the daily doses of levadopa taken by PD patients included in this study were not recorded. Therefore, potential associations between daily levodopa doses and other variables assessed by questionnaire could not be evaluated. In future studies with larger sample sizes, OSS-TR needs to be evaluated before and after treatment in PD patients to determine its minimal clinical importance and responsiveness.

## Conclusion

The OSS-TR is a valid and reliable assessment instrument for the evaluation of sitting balance in Turkish-speaking PD patients. It is concluded that a multidimensional assessment of sitting balance in PD patients with OSS-TR may provide significant benefits in clinical and research settings.

### Ethics statement

Ethics committee approval of this study was obtained from Kırşehir Ahi Evran University Ethics Committee (Number: 2024-12/93).

## Data Availability

Data availabilityThe datasets generated during and/or analysed during the current study are not publicly available due to privacy reasons, but are available from the corresponding author on reasonable request.
